# Does the applied polytrauma definition notably influence outcome and patient population? – a retrospective analysis

**DOI:** 10.1186/s13049-017-0400-2

**Published:** 2017-08-31

**Authors:** Stephan Frenzel, Philipp Krenn, Thomas Heinz, Lukas Leopold Negrin

**Affiliations:** Department of Trauma Surgery, Medical University of Vienna, Waehringer Gürtel 18-20, 1090 Vienna, Austria

**Keywords:** Polytrauma, Polytrauma definitions, Comparison of definitions, Berlin definition, Patient population, Mortality rate, Outcome evaluation

## Abstract

**Background:**

Although the term “polytrauma” has been in use for decades, no generally accepted definition exists. The aim of this study was to demonstrate that different polytrauma definitions applied to a specific patient population result in diverse subgroups of individuals, who in turn present a varying outcome.

**Methods:**

All patients (≥18 years) treated at our level I trauma center within a time period of three years were classified according to 11 selected polytrauma definitions and included in our study, if they were rated “polytraumatized” by at least one of these definitions. All patients, who met the criteria of a certain definition, were combined to the relevant definition-positive group, thus resulting in 11 patient subgroups. Their demographic data (number of patients, mean patient age, mean Injury Severity Score value, median number of ventilator days, median length of stay at the intensive care unit and at the hospital, mortality rate and odds ratio) were statistically compared.

**Results:**

Three hundred seventy-five patients (73% male) with a mean age of 47 years met the inclusion criteria and were allocated to the relevant subgroups; their patient number varied from 55 to 346 and their mean Injury Severity Score value ranged from 4 to 75. Not surprisingly, all examined parameters were subject to variations. Whereas most definition-positive groups showed a mortality rate of about 21% to 30%, 18% of the individuals, who met the criteria according to Blacker, and 40% of the polytrauma victims according to Schalamon died. The Pape 1-, Schalamon-, and Berlin-positive groups presented a significant odds ratio with regard to mortality that considerably exceeded 1.

**Discussion:**

A polytrauma definition can only be a reliable tool in classifying trauma victims if it provides a significant odds ratio with regard to mortality that considerably exceeds 1 and if it succeeds in capturing patients with multiple severe injuries and a higher mortality rate without reducing the number of polytraumatized patients to a not representatively small number.

**Conclusions:**

Solely the Berlin definition resulted in a patient number reflecting clinical reality, thus enabling a transparent evaluation of treatment results provided by different institutions and allowing objective comparison of published studies.

## Background

Worldwide, trauma is an important public health concern. Every year more than 5 million people die from non-intentional traumatic injuries, homicide and suicide, representing 9% of global deaths [1]. Additionally, 20 to 50 million people suffer non-fatal injuries, many of them resulting in disability [2]. The severity of trauma is classified by the Abbreviated Injury Scale (AIS) that was introduced in 1969 and most recently modified in 2008 [3]. In order to quantify the overall severity of multiple injured patients the Injury Severity Score (ISS) was presented in 1974 [4]. It is calculated as the sum of the squares of the highest AIS code in each of the three most severely affected body regions.

Following the initial definition by Tscherne [5] et al, it is generally standard that “polytrauma” denotes multiple and severely injured patients with a high risk of morbidity and mortality, which is higher than the sum of morbidity and mortality of the individual injuries, and with a high risk of cost consuming therapeutic demands. Although this term has been in use for many decades, there is no consensus among clinicians and researchers about the most appropriate definition to identify multiple injured patients with a substantial systemic inflammatory response [6]. A review article, published in 2009, revealed 47 different definitions [7]. They focused on the number of injuries, body regions or organ systems involved [8, 9], pattern or mechanism of injury [10, 11], injuries representing a threat to life [12, 13], ISS [14, 15], and threat to life plus ISS [16]. Since then, even further definitions have been published, wholly or partially based on the AIS [17, 18]. At present, the ISS is used as a standard classification of multiple trauma in the United States, many European countries, and Australia [17].

Undoubtedly, a generally accepted polytrauma definition is crucial for scientific and clinical reasons such as the adequate comparison of results that are presented by different research groups as well as for the benchmarking of care. Polytrauma management is highly resource intensive. Due to the present financial situation, economic aspects gain in relevance. In many countries trauma networks are established and specialized centers are graded in different levels according to their infrastructural capacities and a required minimal caseload of polytraumatized patients [19]. Furthermore, critical quality management including objective measurable parameters such as hospital costs per inpatient day, length of stay at the intensive care unit (ICU), or mortality rate has to be based on a clearly specified patient population. As a result of missed standardization, data in regard to mortality rate of multiple injured patients range from 9 to 48% in the previous literature [20–23]. In order to demonstrate that polytrauma definition and case number are closely connected we exemplarily focused on the annual report of the German Trauma Register DGU® [24]. The official inclusion criteria of this database require patient’s admission to the resuscitation room and a subsequent stay at the ICU, resulting in 38,046 recorded patients in the year 2014. Among these individuals 16,843 met the criterion “ISS ≥ 16”. The addition of one physiological risk factor (age ≥70 years, acidosis, unconsciousness, hypotension, coagulopathy) [19] resulted in 9,486 severely injured. This number was reduced to 4,524 by the application of the additional criterion “two body areas with AIS ≥ 3”. Focusing solely on numbers, a high mortality rate of a certain trauma center may be misinterpreted as the result of insufficient treatment and probably lead to budget cuts or restriction of the provided therapy spectrum. In contrast, a low number of in-hospital deaths may cause unjustified benefits or even inappropriate treatment appraisal.

Therefore, the objective of our study was to point out that polytrauma definitions applied to a specific patient population result in different subgroups of individuals, who in turn present a varying outcome in general with great differences in mortality rate and duration of hospitalization in particular.

## Methods

After study approval by our local ethics committee all patients with a minimum age of 18 years, who had been treated at our level I trauma center from January 1, 2009 to December 31, 2011 were classified by 11 well-known and widely used polytrauma definitions [8–18], introduced in Table 1.

**Table 1:** Polytrauma definitions

Dick [11]	1999	Injury to one body cavity (head/thorax/abdomen) plus two long bone and/or pelvic fractures OR injury to two body cavities
McLain [8]	1999	Significant injury (requiring hospital admission and active management) to two or more major organ systems
Pape 1 [14]	2000	ISS ≥ 18
DGU [16]	2002	Injury with ISS ≥ 16 to several physical regions or organ systems, where at least one injury or the combination of several injuries is life-threatening
Schalamon [12]	2003	A life-threatening injury to two or more body regions
Blacker [9]	2004	At least two injuries that involve at least one vital organ (e.g. lung or liver) and necessitate patient admission to a trauma ICU
Sikand [15]	2005	ISS ≥ 16
Zelle [13]	2005	Severely injured patients with two or more severe injuries, with at least one injury or the sum of all injuries being life-threatening
Pape 2 [10]	2006	Injuries of at least two long bone fractures, or one life-threatening injury and at least one additional injury, or severe head trauma and at least one additional injury
Butcher [18]	2012	AIS ≥ 3 in at least two body regions
Berlin definition [17]	2014	AIS ≥ 3 in at least two body regions, ISS ≥ 16 plus one out of five physiologic parameters: hypotension (systolic blood pressure ≤ 90 mmHg), level of consciousness (Glasgow coma scale ≤ 8), acidosis (base excess ≤ -6.0), coagulopathy (international normalized ratio ≥1.4/partial thromboplastin time ≥ 40 s), and age (≥ 70 years)

Apart from two exceptions [16, 17], the name of the first author of the paper including the respective definition was epynomous. An individual was classified “definition-positive” (for example “McLain-positive”), if he/she met the criteria of the relevant definition; otherwise he/she was deemed “definition-negative”. All definition-positive patients according to a given polytrauma classification were combined to a corresponding polytrauma group, therefore resulting in 11 different patient groups. Statistical analysis was performed using IBM SPSS Statistics 20. For each patient age, ISS value, number of ventilator days, length of stay at the ICU (ICU LOS) and overall length of stay (LOS) were collected and combined to a mean and median value, respectively, as appropriate. The Kolmogorov-Smirnov test was used to check for normal distribution. The Chi-square test was applied to analyze significance of odds ratios. A *p*-value of <0.05 was considered significant. Data are presented as mean and standard deviation (SD) for normally distributed parameters, as median and interquartile range (IQR) for skew distributions, or as absolute numbers and percentages.

## Results

**Fig. 1:**
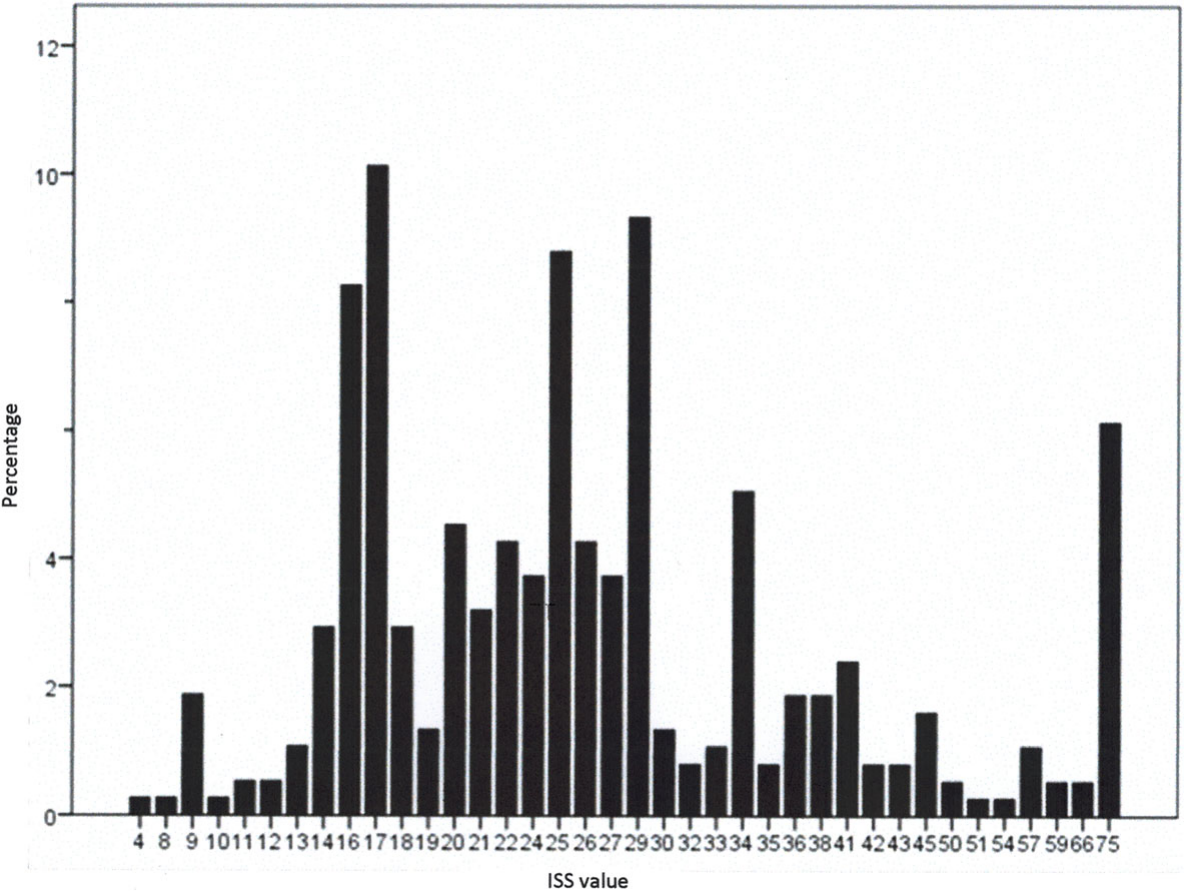
Frequency of ISS values

Over the 3-year study period 375 patients (73% male) with a mean age of 47 ± 19.8 years met the inclusion criteria of at least one definition. Of these individuals, the most frequently injured body region was the head (67%), followed by the thorax (53%) and the upper (45%) and lower extremities (45%). 22% died. The ISS values of 31 trauma victims were lower than 16 (see Fig. 1). Of interest, the lowest value of 4 was found in a patient, who suffered fractures in the diaphysis of two long bones of the same extremity. He was included in the study population according to the definition of Pape 2, not requiring an ISS or AIS cut-off value. In 23 patients the highest ISS value of 75 was calculated. Surprisingly, 133 patients (35.5%) did not spend a single day at the ICU; 111 patients (29.6%) were treated there for one to 10 days. Table 2 illustrates relevant data referring to the different definition-positive groups. Patient numbers varied widely.

**Table 2:** Demographic data

	McLain	Dick	Pape 1	DGU	Blacker	Schala-mon	Sikand	Zelle	Pape 2	Butcher	Berlin
Patient number/percentage	15842	18449	27874	249 66.5	17546.5	5514.5	346 92.5	174 46.5	223 59.5	17446.5	130 34.5
Mean age (years)/SD	44.2 18.4	43.7 18.4	46.8 20.1	45.3 19.2	42.7 18.4	43.321.3	46.720	43.718	44.319	4317.9	45.319
Mean ISS/SD	3415.5	32.5 16	3315.5	3214.5	3011.5	4815	3015	3515.5	32.516	3515	37.518
Median ventilator days/IQR	31-8	30-9	20-8	20-8	52-11	41-9	20-8	30-9	20-9	31-8	42-9
Median ICU LOS (days)/IQR	104-22	103-22	92-21	90-20	136-23	189-28	70-19	114-23	90-22	81-20	113-21
Median overall LOS (days)/IQR	3319-65	3215-63	2915-59	3015-61	3222-63	6125-85	2514-48	3620-69	3216-62	2610-62	308-63
Mortality rate (%)	21.5	22.5	26	21	18	40	23	22.5	23	22.5	30

Noteworthy, all individuals diagnosed as polytrauma victims according to Schalomon were also rated polytraumatized by at least five other classifications. Whereas most definition-positive groups showed a mortality rate of about 21% to 30%, 18% of the individuals, who met the criteria according to Blacker, and 40% of the polytrauma victims according to Schalamon died. Furthermore, there were substantial differences in most corresponding data between the Schalamon-group and the other groups (Table 2). Table 3 displays the odds ratios for mortality. Solely the ratios referring to the Pape 1-, Schalamon-, Sikand- and Berlin-groups significantly exceeded 1, indicating that for those people, who had been classified definition-positive, the odds to die was two to four times higher, respectively, compared to the relevant definition-negative group.

**Table 3:** Odds ratios referring to mortality

	OR	95% CI	*p*-value
McLain	0.965	0.588–1.586	0.990
Dick	1.049	0.643–1.712	0.947
Pape 1	3.041	1.499–6.168	0.002
DGU	0.905	0.541–1.512	0.802
Blacker	0.629	0.381–1.039	0.090
Schalamon	2.889	1.573–5.307	<0.001
Sikand	4.060	0.945–17.446	0.072
Zelle	1.062	0.650–1.734	0.910
Pape 2	1.157	0.700–1.915	0.658
Butcher	1.061	0.650–1.734	0.910
Berlin	2.013	1.222–3.316	0.008

## Discussion

To our knowledge this is the first study investigating the influence of 11 different, well-known and widespread polytrauma definitions on patient population and outcome of a single level I trauma center. Depending on the applied definition we revealed widely varying numbers of polytraumatized patients providing major differences in mortality rates and mean ISS values. Unfortunately, some definitions are not spelled out clearly and their concrete application had to be clarified. According to McLain a significant injury “requires hospital admission and active management” [8]. This specification leaves room for subjective interpretation especially in regards to “active management”. Furthermore, the term “life-threatening” is an integral part of four of the applied definitions, although never explained in detail [10, 12, 13, 16]. Because vague phrases are subject to high inter-observer variation comparison of results is difficult in terms of reliability and accuracy. Schalamon’s definition inherently targets the critically injured [12], thus resulting in a small number of patients, presenting a high mortality rate of 40%. The polytrauma definition of Sikand, however, resulted in a more than six time larger patient group with a mortality rate of 23%. The remarkably high (yet statistically not significant) odds ratio referring to mortality has to be interpreted with caution due to the fact that the Sikand-negative group was particularly small, comprising solely individuals with ISS values lower than 16. The low lethality rate and the low ISS values in the group classified according to Blacker were mainly caused by the fact that patients’ admission to the ICU is part of the definition [9]. Because individuals, who died at the trauma resuscitation room had to be excluded polytrauma victims according to Blacker do not represent the actual patient population of a level I trauma center. The same applies to polytrauma definitions solely based on cut-off values (e.g. ISS ≥ 16 or ISS ≥ 18) because they also include individuals with a severe monotrauma (e.g. head trauma) [18]. Undoubtedly, applying such a definition impedes the inter-institutional comparison of mortality and resource utilisation as it fails to discriminate those centers, which treat the most severely injured of the trauma patient population, and eventually leads to an inappropriate budget distribution and use of infrastructural capacities [18]. Using higher ISS cut-off values (e.g. ISS > 20 or ISS > 25) results in an increased specificity but excludes patients with two injuries rated AIS = 3. In general, the exclusive use of an anatomical score ignores the physiological aspects of polytrauma, which are supposed to pose an extremely important additional factor in polytrauma rating [25, 26]. Of the 11 definitions scrutinized here, solely the Berlin definition includes five independent physiologic parameters, which were chosen by a panel of international experts after extensive research. For evaluation of parameter levels all polytraumatized rated patients of the nationwide trauma registry of the DGU were used. Cut-off values for each specific parameter of the Berlin definition were then calculated in such a way that the trauma victims provided a mortality rate of 30%, which is twice as high as the mortality rate of the patient population with an ISS of 16 points [17].

To our opinion a polytrauma definition can only be a reliable tool in classifying trauma victims if it provides a significant odds ratio with regard to mortality that considerably exceeds 1 and if it succeeds in capturing patients with multiple severe injuries and a higher mortality rate without reducing the number of polytraumatized patients to a not representatively small number.

Limitations of this study include the fact that it primarily focuses on mortality, also showing differences in patient number and age, ISS value, number of ventilator days, ICU LOS, and LOS between the 11 definition-positive groups. However, important factors in multiple injured patients such as the risk for major inflammatory complications and multiple organ failure as well as the need of a well-trained multidisciplinary team have not been taken into consideration.

## Conclusions

The Berlin definition reflects clinical reality, thus enabling a transparent evaluation of treatment results provided by different institutions, which is indispensable for adequate and reliable benchmarking of care and outcomes.

## Abbreviations

AIS: Abbreviated Injury Scale
ICU: Intensive care unit
ISS: Injury Severity Score
